# Influence of Thickness in the Color Masking Ability of Different Types of Computer‐Aided Design and Manufacturing Ceramics

**DOI:** 10.1002/cre2.70438

**Published:** 2026-08-19

**Authors:** Seyede Mohaddeseh Hosseini Kalej, Behnaz Esmaeili, Ghazaleh Ahmadi Zenouz, Hemmat Gholinia

**Affiliations:** ^1^ Student Research Committee Babol University of Medical Sciences Babol Iran; ^2^ Dental Materials Research Center, Health Research Institute Babol University of Medical Sciences Babol Iran; ^3^ Oral Health Research Center, Health Research Institute Babol University of Medical Sciences Babol Iran

**Keywords:** ceramic, masking, translucency

## Abstract

**Objectives:**

Tooth discoloration is a major esthetic concern in cosmetic dentistry. This study investigated the color‐masking ability of four CAD/CAM ceramics and the influence of material type and thickness on their optical properties.

**Materials and Methods:**

A total of 120 A1‐shade CAD/CAM ceramic specimens were prepared from Vitablocs Mark II, Vita Suprinity PC, Celtra Duo, and Ceramill Zolid FX (*n* = 30 per material). Each material was fabricated in three thicknesses (0.5, 1, and 1.5 mm; *n* = 10). Color‐masking ability was evaluated using A1 and A5 composite substrates. Translucency of 1‐mm specimens was assessed against black and white backgrounds. CIELab values were measured using a spectrophotometer, and color differences (Δ*E* ^∗^_ab_, Δ*E*_00_) and translucency parameters (TP, TP_00_) were calculated. Data were analyzed using one‐way and two‐way ANOVA and Tukey's test (*p* < 0.05).

**Results:**

Ceramic thickness significantly influenced color differences, with thicker ceramics exhibiting lower color differences and greater masking ability (*p* < 0.001). Significant differences in translucency parameters and color difference values were also observed among the tested ceramics (*p* < 0.001). Zolid FX at 1.5 mm showed the highest color‐masking ability, whereas Vita Suprinity at 0.5 mm showed the lowest.

**Conclusions:**

The study showed that increasing the thickness of ceramic materials significantly improved their ability to mask underlying colors, highlighting the importance of material depth in achieving esthetic results. Furthermore, it was found that a thickness of at least 1 mm is required for zirconia ceramics (ZF) and 1.5 mm for VM ceramics to effectively mask an A5 background shade. In contrast, Celtra (CD) and Vita Suprinity (VS) ceramics did not demonstrate adequate color masking effectiveness at any of the tested thicknesses, indicating their drawbacks for clinical scenarios where optimal esthetic outcomes are essential.

## 1 Introduction

As society increasingly demands esthetic appeal, particularly in the form of attractive smiles and bright white teeth, addressing tooth discoloration has gained significant importance in cosmetic dentistry. Given the varying degrees of tooth discoloration, multiple treatment approaches are available, such as bleaching for both vital and nonvital teeth, micro‐abrasion, porcelain and composite veneers, porcelain crowns, and even a combination of the aforementioned methods (Galiatsatos and Galiatsatos 2024; Antwan 2024).

All‐ceramic restorations have become popular due to their natural appearance, offering qualities such as translucency and fluorescence, along with biocompatibility, chemical stability, high compressive strength, long‐lasting color retention, excellent post‐adhesive bond strength, and thermal expansion characteristics similar to natural tooth structure (Alayad et al. 2021).

The final appearance of ceramic restorations is determined by a variety of factors, including the type of ceramic used, its composition and microstructure, translucency and optical characteristics, layering techniques, surface texture, thickness, the color and opacity of the bonding agents utilized, and the underlying tooth color (Iravani et al. 2020; Giordano II 2022). Increasing the thickness of a restoration can provide better coverage for discolored teeth. However, this could also lead to over‐contouring. On the other hand, excessive tooth reduction may compromise the bond strength of the ceramic to the tooth or damage the pulp vitality (Giordano II 2022; Kandil et al. 2019).

Computer‐aided design and manufacturing (CAD/CAM) technology has been widely used due to the emergence of new materials, decreased laboratory fabrication time and costs, and improved quality control mechanisms (Ikubanni et al. 2022). VITABLOCS Mark II (VITA Zahnfabrik, Germany) is an example of a monochrome CAD/CAM feldspathic ceramic, comprising feldspathic porcelain particles within potassium alumina silicate glass (Jafari et al. 2018).

Moreover, Zirconia‐reinforced lithium silicate ceramics (ZLS) are an attractive alternative to lithium disilicate ceramics due to their advantageous mechanical and esthetic properties, particularly in high‐demand patients. Vita Suprinity PC (VITA Zahnfabrik, Germany) and Celtra Duo (Dentsply Sirona Restorative, Germany) are two commercially available examples of ZLS products (Rizo‐Gorrita et al. 2018). Recently, advancements in fully stabilized cubic and tetragonal highly translucent zirconia materials have been introduced. These novel materials contain stabilizing oxides and approximately 50% cubic phase, which yields both high translucency and strength. Zirconia laminate veneers crafted from these CAD/CAM ceramics can be produced with a thickness as thin as 0.2–0.3 mm (Zadeh et al. 2018; Vafaei et al. 2019).

Several parameters are used to assess the masking effectiveness of restorative materials. These include the contrast ratio (CR), translucency parameter (TP), and the CIELab color system, which encompasses color difference formulas such as CIELAB (Δ*E*∗_ab_) and CIEDE2000 (Δ*E*_00_) (Basegio et al. 2019). Despite the significance of ceramic thickness in influencing masking ability, limited studies have been conducted in this field. The objective of this study is to examine the color‐masking capability of four different types of CAD/CAM ceramics. The null hypotheses for this research are as follows: ceramic masking ability is independent of its thickness, and translucency is not influenced by the type of ceramic used.

## 2 Materials and Methods

### 2.1 Preparation of Samples

In this study, a total of 120 samples were prepared from four different types of CAD/CAM ceramics, all in A1 shade (Table 1). The sample size was calculated to be 120 participants based on the findings reported by Shadman et al. taking into account the primary outcome measure, a statistical power of 80%, and a 95% confidence level (Shadman et al. 2015). Each type of ceramic consisted of 30 samples, prepared in three different thicknesses: 0.5, 1, and 1.5 mm. This resulted in a total of 12 groups, with each group containing 10 samples (*n* = 10).

**Table 1:** Type of ceramics used in the study.

CAD/CAM ceramic	Material	Shade	Composition	Manufacturer	LOT No.
Vitablocs Mark II (VM)	Feldspathic ceramic	A1	54%–64% SiO_2_, 20%–23% Al_2_O_3_, 6%–9% Na_2_O, 6%–8% K_2_O_6_	VITA, Zahnfabrik, Bad Säckingen, Germany	56670
Celtra Duo (CD)	Fully crystallized Zirconia‐reinforced lithium silicate/phosphate glass ceramic	HT^a^‐ A1	58% SiO_2_, 18.5% Li_2_O, 10.1% ZrO_2_, 5% P_2_O_5_ <10% Pigments	Dentsply Sirona, Charlotte, USA	16005079
Vita Suprinity PC (VS)	Partially crystallized Zirconia‐reinforced lithium silicate glass ceramic	HT‐ A1	56%–64% SiO_2_, 15%–21% Li_2_O, 8%–12% ZrO_2_, <10% Pigments	VITA Zahnfabrik, Bad Säckingen, Germany	50840
Ceramill Zolid FX (ZF)	High translucent Zirconia	0/A1	ZrO_2_ + HfO_2_ + Y_2_O_3_ ≥ 99%, 8.5%–9.5% Y_2_O_3_, Al_2_O_3_ 0.5%≤, Other oxides (except Er_2_O_3_ ≤ 0.1%	Amann GirrbachAG, Koblach, Austria	1907000

^a^ High translucent.

The ceramic samples were prepared through a series of steps to ensure they met the required specifications. Initially, the CAD/CAM ceramics were cut using a Delta Precision Sectioning Machine (Mashhad, Iran) equipped with a continuous water flow to prevent overheating during cutting. The samples were prepared to the dimensions of 12 × 14 × 1.5 mm, 12 × 14 × 1 mm, and 12 × 14 × 0.5 mm, with consideration for the 23.37% shrinkage that occurs during the sintering of zirconia ceramics (ZF), necessitating larger initial sizes.

Following the cutting process, the samples were polished using silicon carbide paper with grits of 400, 600, 800, and 1200 while continuously rinsing with water to achieve the targeted thicknesses of 1.5 ± 0.01, 1 ± 0.01, and 0.5 ± 0.01 mm. The thickness of each sample was measured with digital calipers (Shinwa Digital Caliper, Niigata, Japan), followed by a cleaning process in an ultrasonic bath (Ultrasound Vita‐Sonic II Vita Zahnfabrik, Germany) using distilled water for 10 min, after which the samples were dried with compressed air.

One surface of the VM samples was glazed with VITA AKZENT Plus Glaze Powder (VITA Zahnfabrik, Bad Sackingen, Germany) at 950°C for 10 min in a VITA VACUMAT 6000M furnace (VITA Zahnfabrik, Bad Sackingen, Germany). The CD samples received glazing with Celtra Universal Glaze (Dentsply Sirona Restorative, Germany) at 820°C for 8 min. The VS samples first underwent a crystallization step at 840°C according to manufacturer guidelines, with glazing completed simultaneously for 12 min using VITA AKZENT Plus Glaze LT powder.

The non‐glazed surfaces of the VM, VS, and CD ceramics were etched with a 5% hydrofluoric acid gel (Pulpdent, USA). VM samples were etched for 60 s, VS for 20 s, and CD for 30 s. The residual acid was thoroughly rinsed off, and the samples were cleaned in an ultrasonic bath containing 96% ethyl alcohol for 5 min before being air‐dried. This resulted in a white opaque appearance due to the etching process.

In preparation for bonding, Silane (Pulpdent, USA) was applied to the etched surfaces of the ceramics using a micro‐brush; after allowing it to sit for 1 min, the excess was removed by air drying. The ZF samples underwent a sintering process in a KFP Dental Auto Sinter 1650 furnace (Kousha Fan Pars, Iran), where they were heat‐treated at 1450°C for 10 h, achieving the desired translucency and mechanical strength. Sample thickness was verified by caliper (Shinwa Digital Caliper, Niigata, Japan).

**Diagram 1:**
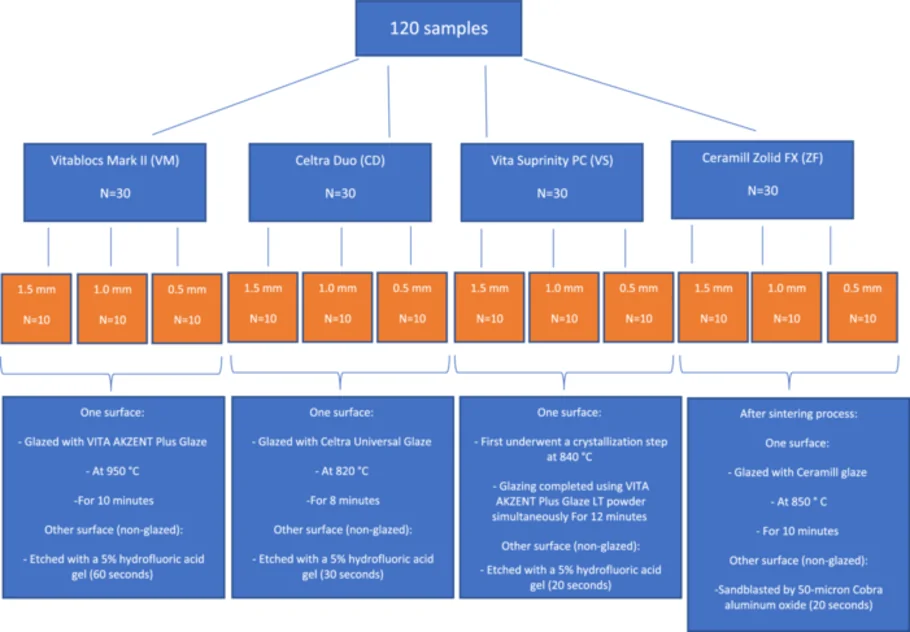
Summarize of the study design, groups and sample preparation.

Finally, one surface of the ZF samples was glazed using Ceramill glaze (Amann GirrbachAG, Koblach, Austria) at 850°C for 10 min in a KFP Auto Therm300 furnace (Kousha Fan, Iran). To enhance their bonding capabilities, the unglazed surfaces of the ZF samples were sandblasted for 20 s using 50‐micron Cobra aluminum oxide particles (Renfert, Hilzingen, Germany) in FineBlast (KFP Dental, Tehran, Iran); the tip of the sandblaster was positioned at a distance of 10 mm from the samples and sandblasting was performed under two bar pressure (Diagram 1, 1).

### 2.2 Preparation of Composite Background

Vit‐L‐essence composite (Ultradent, South Jordan, USA) was used to prepare two backgrounds in A5 and A1 shades. The A5 background was used to simulate dark infrastructure, and the A1 background was the control. To make each of the backgrounds, a Plexiglas mold with the dimensions 14 mm length, 12 mm width, and 4 mm height was filled with composite in two layers of 2 mm each and the exposure of each layer was cured using an LED light curing unit (Valo, Ultradent, South Jordan USA) with an intensity of 1000 mW/cm^2^ for 20 s.

A layer of mylar strip and a glass slab were placed on top of the composite to create an even surface. The head of the curing device was adjusted to be in contact with the glass slab. After curing, the mylar strip and glass slab were removed, and the composite was slowly removed from the mold and cured for another 40 s from the bottom surface, then polished with 400–2000 grit silicon carbide papers.

Resin cement (Choice 2, Bisco, Schaumburg, IL, USA) was used in this study. To minimize the effect of cement color on the results, the translucent type of cement was used. A layer of 0.1‐mm‐thick cement was cured on both composite background substrates so that each of the composite backgrounds was placed inside a mold with dimensions of 14 mm (length) × 12 mm (width) × 4.1 mm (height), and cement was applied into the remaining space. Mylar strips and glass slabs were placed on uncured resin cement, and the cement was pressed with finger pressure. The cement was cured using a Valo light curing unit for 40 s.

### 2.3 Measurements of Color Differences

**Figure 1:**
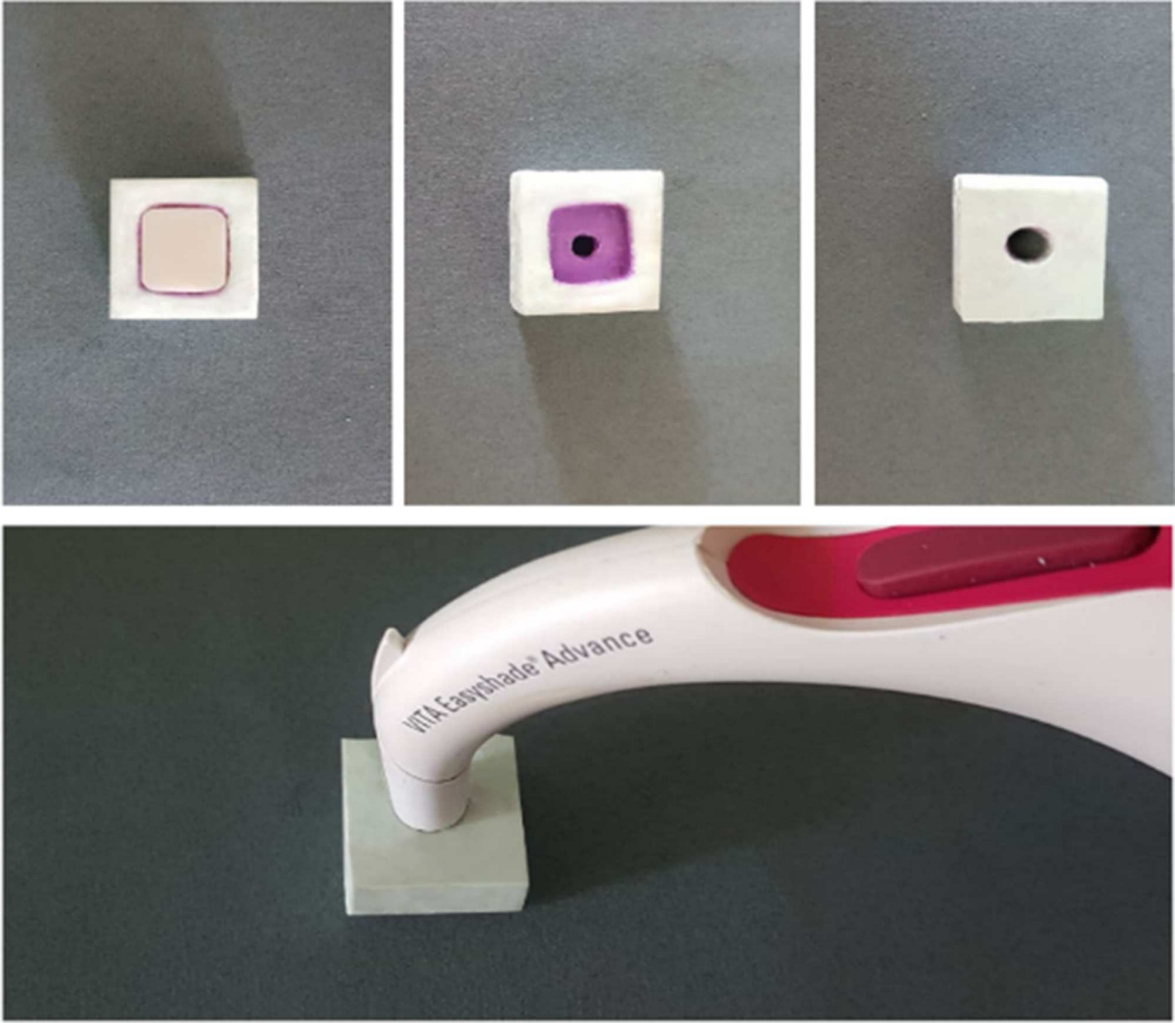
Silicone mold and Vita Easyshade Advance.

Measurements were performed on A1 (L* = 84.8, a* = −2, b* = 14.1) and A5 (L* = 66.2, a* = −0.7, b* = 26.1) composite backgrounds using a spectrophotometer (Vita Easyshade Advance; Vita Zahnfabrik, Bad Sackingen, Germany) by a skilled dental student. The device was set to single‐tooth mode. Calibration of the device was performed before measurement by placing the probe tip of the device on a special calibration plate. The probe tip was then placed in the middle of the ceramic specimens, creating a 90‐degree angle with the surface of the samples. To eliminate the effect of surrounding light and to reproduce identical testing parameters for all groups, a silicone mold (Speedex Putty and light body, Colténe/Whaledent AG, Altstätten, Switzerland) was made around the head of the Vita Easyshade and ceramic sample (Figure 1, 1). Samples were then placed on A1 and A5 backgrounds, and the parameters L*, a*, b*, C*, and *h*° were measured three times for each sample on each composite background. The mean values were used to calculate the quantitative color difference value (Δ*E* ^∗^_ab_) according to the following formula:

$$\Delta E{*}_{\mathrm{ab}}=\sqrt{{\left(\Delta L*\right)}^{2}+{\left(\Delta a*\right)}^{2}+{\left(\Delta b*\right)}^{2}},$$

$$\Delta L\;*\;=L{*}_{T}-L{*}_{C,}\Delta a\;*\;=a{*}_{T}-a{*}_{C},\Delta b\;*\;=b{*}_{T}-b{*}_{C}.$$

*T* parameters represent the test group (background A5).

*C* parameters represent the control group (background A1).

Calculation of Δ*E*_00_ in the CIEDE2000 color difference system was performed based on the following formula:

$${\Delta E}_{00}=\sqrt{{\left(\frac{\Delta L\prime }{K_{L}S_{L}}\right)}^{2}+{\left(\frac{\Delta C\prime }{K_{C}S_{C}}\right)}^{2}+{\left(\frac{\Delta H\prime }{K_{H}S_{H}}\right)}^{2}+R_{T}(\frac{\Delta C\prime }{K_{C}S_{C}})(\frac{\Delta H\prime }{K_{H}S_{H}})},$$

ΔH′,ΔC′,ΔL′ show the difference in degree of brightness, chroma, and hue of sample on A1 and A5 backgrounds, respectively. RT (rotation function) shows the interaction between the difference in chroma and hue in the blue range. SL, SC, and SH are degrees of lightness, chroma, and hue, respectively, based on weight functions. KL, KC, and KH are parametric factors that are adjusted based on different observational indices.^3^ In the present study, these parametric coefficients are considered one (KL = 1, KC = 1, KH = 1).

Δ*E*∗_ab_ and Δ*E*_00_ values were compared with the perceptibility threshold (PT: Δ*E*∗_ab_ = 1.22, Δ*E*_00_ = 0.81) and acceptability threshold (AT Δ*E*∗_ab_: = 2.66, Δ*E*_00_ = 1.77) (Ikubanni et al. 2022).

### 2.4 TP

Translucency was evaluated in samples with a thickness of 1 mm. Translucency measurements were performed on two black‐and‐white backgrounds. For each sample, the parameters L*, a*, b*, *c*, and *h* were measured using Vita Easyshade Advance three times, and their means were used to calculate the TP in the following formulas:

$$\text{TP}=\sqrt{{\left(\Delta L*\right)}^{2}+{\left(\Delta a*\right)}^{2}+{\left(\Delta b*\right)}^{2}},$$

$$\Delta L\;*\;=L{*}_{B}-L{*}_{W},\Delta a\;*\;=a{*}_{B}-a{*}_{W},\Delta b\;*=b{*}_{B}-b{*}_{W}.$$

*B* represents the parameters over the black background, and *W* represents the parameters over the white background.

TP was also calculated in CIEDE2000 using the following formula:

$$\text{TP}00=\sqrt{{\left(\frac{\Delta L\prime }{K_{L}S_{L}}\right)}^{2}+{\left(\frac{\Delta C\prime }{K_{C}S_{C}}\right)}^{2}+{\left(\frac{\Delta H\prime }{K_{H}S_{H}}\right)}^{2}+R_{T}(\frac{\Delta C\prime }{K_{C}S_{C}})(\frac{\Delta H\prime }{K_{H}S_{H}})},$$

ΔH′,ΔC′,ΔL′ show the difference in degree of brightness, chroma, and hue, respectively, over the white and black backgrounds. Other parameters were considered as the TP_00_ formula.

### 2.5 Statistical Analysis

Statistical analysis was performed using SPSS 25 (IBM, SPSS Inc.). TP analysis of different ceramics was performed using one‐way analysis of variance (ANOVA) and Tukey's post hoc test. To evaluate the color‐masking ability (Δ*E*∗_ab_ and Δ*E*_00_) in different types of ceramics with different thicknesses, two‐way ANOVA and then one‐way ANOVA and Tukey's HSD post hoc tests were used. The comparison of ΔE*_ab_ and Δ*E*_00_ data with PT and AT was performed using one sample and paired *t*‐tests. *p* < 0.05 was considered statistically significant.

## 3 Results

Normality of the data distribution was assessed using the Shapiro–Wilk test. Since ΔE00 values showed a normal distribution, comparison of color difference among the three ceramic thicknesses (0.5, 1.0, and 1.5 mm) within each ceramic type was performed using one‐way ANOVA, followed by Tukey's post‐hoc test for pairwise comparisons when appropriate. In addition, although ceramic thickness had only three discrete levels, it represents an ordered quantitative variable. Therefore, following the reviewers' recommendation, a Pearson correlation analysis was conducted to determine the direction and strength of the linear relationship between ceramic thickness and Δ*E*_00_ and Δ*E*_ab_. This analysis was performed separately for each ceramic material to better illustrate how thickness influences masking ability in each group.

Two‐way ANOVA showed that the effect of ceramic type (*p* < 0.001), the effect of thickness (*p* < 0.001), and also the interaction between ceramic type and thickness on Δ*E*_00_, ΔE*_ab_ values (*p* < 0.001) were significant.

**Table 2:** The Mean of Δ*E*_ab_ and Δ*E*_00_ in different types and thicknesses of CAD‐CAM ceramics.

CAD/CAM ceramic	0.5 mm		1 mm		1.5 mm		*p* value
	Δ*E*_00_	Δ*E*_ab_	Δ*E*_00_	Δ*E*_ab_	Δ*E*_00_	Δ*E*_ab_	
VM	2.33 (0.15)^aA^∗	1.48 (0.09)^a1^∗	3.54 (0.20)^a^	2.2 (0.12)^a2^	6.37 (0.57)^aC^	4.13 (0.39)^a3^	> 0.001
CD	2.59 (0.34)^aA^	1.72 (0.22)^b1^	3.68 (0.59)^aB^	2.34 (0.39)^a2^	6.85 (0.43)^aC^	4.49 (0.29)^a3^	> 0.001
VS	2.99 (0.39)^bA^	2.05 (0.26)^c1^	4.98 (0.53)^bB^	3.42 (0.4)^b2^	8.52 (0.81)^bC^	5.88 (0.6)^b3^	> 0.001
ZF	1.89 (0.31)^cA^∗	1.16 (0.17)^d1^∗	2.59 (0.23)^cB^	1.58 (0.15)^c2^∗	3.84 (0.16)^cC^	2.35 (0.11)^c3^	> 0.001
*p*‐Value	> 0.001		> 0.001		> 0.001		

*Note:* CD: Celtra Duo, VM: VITABLOCS Mark II, VS: VITA SUPRINITY PC, ZF: Ceramill Zolid FX.

As seen in Table 2, according to the one‐way ANOVA and post hoc Tukey's test, the mean values of ΔE*_ab_ and Δ*E*_00_ between different thicknesses of each ceramic were significantly different (*p* < 0.001). As the thickness of the samples increased, both Δ*E*_00_ and ΔE*_ab_ values decreased (*p* < 0.001). The highest and lowest color masking were observed in ZF samples with a thickness of 1.5 mm and VS with a thickness of 0.5 mm, respectively. One‐way ANOVA showed significant differences in the mean ΔE*_ab_ and Δ*E*_00_ values between ceramics at the same thickness (*p* < 0.001). Post Hoc Tukey's test revealed significant differences between the values in each pair of ceramics except VM and CD ceramics at all thicknesses for ΔE*_ab_ and at 1 and 0.5 mm for Δ*E*_00_ (*p* < 0.05).

Different small letters in each column indicate a statistical difference (*p* < 0.05). Capital letters and different uppercase numbers in each row indicate a statistically significant difference in the values of ΔE*_ab_ and Δ*E*_00_ in different thicknesses of each ceramic, respectively (^∗^indicate Δ*E* values less than acceptability thresholds (AT Δ*E*∗_ab_: = 2.66, Δ*E*_00_ = 1.77).

Using a one‐sample t‐test, it was found that ΔE*_ab_ and Δ*E*_00_ values were significantly lower than AT in both VM and ZF ceramics with a thickness of 1.5 mm. In the case of Δ*E*_00_, values were also smaller than AT at the 1 mm thickness of ZF ceramic (AT Δ*E*∗_ab_: = 2.66, Δ*E*_00_ = 1.77).

Considering Δa*, Δb*, and ΔL* values in different thicknesses of VM, VS, and ZF ceramics, differences in the mean Δb* values were only significant in VM and versus However, significant differences in the mean Δa* and ΔL* were found in all four types of ceramics, and with increasing thickness, ΔL* values decreased significantly in all groups.

Pearson's correlation test showed a strong negative relationship between color difference values (ΔE*_ab_ and Δ*E*_00_) and ΔL*: Pearson correlation coefficients (*r*) were 0.992 and 0.948, respectively. A moderate positive correlation was noted between the ΔE*_ab_, Δ*E*_00_, and the Δa* (*r*: 0.450 and 0.411, respectively). However, no significant relationship was shown between ΔE*_ab_, Δ*E*_00_, and Δb*.

Pearson correlation analysis demonstrated a strong and statistically significant positive correlation between thickness and Δ*E*_00_ and Δ*E*_ab_ for all ceramic types. The correlation coefficients for each ceramic and Δ*E*_ab_ and Δ*E*_00_ are shown below:

Vitabloc Mark II: Δ*E*_ab_ (*r* = 0.955, *p* < 0.001) Δ*E*_00_ (*r* = 0.947, *p* < 0.001).

Celtra Duo: Δ*E*_ab_ (*r* = 0.933, *p* < 0.001) Δ*E*_00_ (*r* = 0.924, *p* < 0.001).

Suprinity: Δ*E*_ab_ (*r* = 0.959, *p* < 0.001) Δ*E*_00_ (*r* = 0.953, *p* < 0.001).

Zolid FX: Δ*E*_ab_ (*r* = 0.946, *p* < 0.001) Δ*E*_00_ (*r* = 0.946, *p* < 0.001).

These results indicate that changes in ceramic thickness are strongly linked to changes in color masking performance across all ceramic systems.

Table 3 shows the TP and TP_00_ values of each CAD/CAM ceramic with a thickness of 1 mm. Regarding the TP, there were significant differences in mean TP and TP_00_ values in different ceramics. A pair‐wise comparison of groups with Tukey's post‐hoc test revealed significant differences between all groups except for VM and CD.

**Table 3:** The Mean of TP and TP_00_ values in different types of CAD/CAM ceramics in 1 mm thickness.

Translucency parameter	VM	CD	VS	ZF
TP	10.90 (0.78)^A^	11.25 (0.83)^A^	14.73 (0.97)^B^	7.47 (0.3)^C^
TP_00_	6.81 (0.47)^A^	7.3 (0.5)^A^	10.3 (0.64)^B^	4.74 (0.21)^C^

*Note:* Different uppercase letters (A–C) within each row indicate statistically significant differences (*p* < 0.05) in the translucency index of the different ceramic materials.

## 4 Discussion

In this study, the translucency and color‐masking ability of four types of CAD‐CAM ceramics were evaluated. The findings showed that the thickness of the ceramic had a significant effect on masking the color of the underlying substrate, and by increasing the thickness of the ceramics (from 0.5 to 1.5 mm), the color‐masking ability was enhanced. Thus, our first null hypothesis was rejected. Furthermore, the present study showed significant differences in TP and TP_00_ values of different CAD‐CAM ceramics. Accordingly, our second null hypothesis, which stated that the translucency of the substance is not affected by the type of substance, was also rejected.

Restoring discolored anterior teeth is a clinical challenge for most dentists. One of the most important factors that determines the final color of the restoration is the choice of ceramic material. Among the aspects to consider that affect the choice of ceramic material in discolored teeth is the ability to mask (Kahler 2022).

In the current investigation, an A5 shade composite was used to replicate discolored teeth, and the ability of various ceramic specimen thicknesses to mask this discoloration was assessed by measuring ΔE*_ab_ and Δ*E*_00_ values. Nonetheless, it is important to acknowledge the limitations of the ISO standard; the stark contrast between the white and black backgrounds used to determine the TP might lead to color differences that exceed those typically observed in routine dental cases, such as non‐vital discolored teeth or metallic substructures (Basegio et al. 2019). The findings of this study suggested that ceramics with a thickness of 1 mm exhibited TP and TP_00_ values that were greater than the Δ*E*∗_ab_ and Δ*E*_00_ values of the same samples on both A5 and A1 substrates, and even surpassed those of the all‐ceramic restoration (AT). Consequently, according to ISO standards, none of the tested samples proved capable of adequately masking the discolored substrate. However, when these samples were applied over an A5 composite substrate to reflect clinical discoloration settings, 1.5 mm‐thick ZF and VM samples demonstrated Δ*E*∗_ab_ and Δ*E*_00_ values that fell below the threshold of AT, whereas the CD and VS ceramics failed to mask the A5 background at any thickness. In line with current findings, Bacchi et al. suggested that ZLS ceramic, despite being 1.8 mm thick, was not able to mask the substrate shade of A3.5, C4, silvery, and coppery (Bacchi et al. 2019). Moreover, Nikanjam et al. reported that the minimum thickness of ZLS ceramic for ideal masking of nonprecious gold and A3 backgrounds were 1.5 and 2 mm, respectively, whereas B1 and D2 could be covered by a 1 mm thickness (Nikanjam and Tayebi 2023).

Furthermore, the findings of this study showed a strong association between ΔL* and Δ*E*. In agreement with these findings, another study reported that compared to other parameters, ΔL* had the greatest effect on Δ*E*, and the value of L* decreased when placed on a dark background. The black and gray backgrounds reduced the value of b*, while its effect on a* was unclear (Tabatabaian et al. 2017). Moreover, Jiang et al. suggested that ΔL*, Δb*, and Δ*E*_00_ are remarkably associated with patient satisfaction (Nw et al. 2024). On the other hand, the current investigation reported a positive correlation between translucency and Δ*E* values. The VS ceramics exhibited the highest translucency and color difference values, while the ZF ceramics demonstrated the lowest. These findings are consistent with those reported in prior research (Alayad et al. 2021; Bacchi et al. 2019).

Previous studies have reported that the CIEDE2000 evaluates color differences more accurately than the CIELAB system (Pérez et al. 2022). However, in the present study, no significant difference was observed between the two methods.

The results of the present study showed that TP and TP_00_ values were highest in VS and lowest in ZF. Similar results were reported in a study by Carneiro et al. in which translucent zirconia ceramic (Zirkonzahn Prettau) had the lowest translucency and VS had the highest translucency compared to Vita Mark II and IPS e.max Press ceramics (Carneiro et al. 2018). Similarly, Gunal et al. showed that Prettau Anterior was less translucent than VM and VS glass ceramics (Gunal and Ulusoy 2018).

Translucency in ceramic restorations depends on various factors such as crystal structure, particle size, pigments, volume and distribution of defects, and porosity (Wang et al. 2023; Juntavee et al. 2025). Large crystal particles give high strength to ceramics while reducing translucency (Ragab et al. 2020).

Zolid FX is a super high translucent zirconia with an increased amount of stabilizing oxides in its structure, which results in the full cubic stabilized monolithic zirconia formation with optical isotropic properties that reduce the scattering of light between particles. In addition, lower amounts of alumina and relatively small particle size in this ceramic result in higher translucency factors for this group of new zirconia compared to previous generations (Elsaka 2019; Marak et al. 2024). However, the low translucency of translucent zirconia compared to other glass ceramics in our study can be explained by the large amounts of crystal particles and the lack of a glass phase that interferes with the passage of light and its reflection (Bacchi et al. 2019).

Contrary to the findings of our study, an investigation reported that VM was more translucent than VS (Alzahrani et al. 2022). The difference in the translucency of VS blocks used, which was highly translucent in the present study and translucent in their study could be a justification for this discrepancy.

Our findings concur with the results of Choi et al., in which no significant difference was observed in the TP values of high‐translucent CD and VM (Choi et al. 2021). Contrary to our results, Juntavee et al. stated higher translucency of VM than CD, which could be due to the use of low translucent CD blocks in that study (Juntavee et al. 2025). The presence of fewer lithium metasilicate crystals in the pre‐crystallized phase of HT materials and, consequently, less light scattering compared to LT types could be one reason for their translucency difference (Juntavee et al. 2025; Sheibani et al. 2024).

In the present study, the higher translucency of VS compared to CD was similar to the results of previous studies (Alayad et al. 2021). In contrast, Wu et al. suggested that VS samples had lower translucency and higher opalescence compared to the other groups (Wu et al. 2023). The sintering process after milling may cause changes in crystal size and structure which leads to different translucency and opalescence among the materials (Nejatidanesh et al. 2020). Although VS and CD are both zirconia‐reinforced lithium silicate ceramics, the difference in their translucency could be due to variations in the composition and the size of Metasilicate particles which is reported 1 μm in CD and 0.5 μm in VS (Zarone et al. 2021).

Cement shade is an important factor affecting the final color of a restoration. In the present study, a translucent shade was selected so as not to alter the test results. An additional limitation of the present study was that flat, non‐anatomical ceramic samples were studied to attain a controlled color measurement process. Hence, it is suggested that in future investigations, the effect of cement shade and different ceramic thicknesses on the color masking of ceramic restorations on different backgrounds be studied.

## 5 Conclusion

The study showed that increasing the thickness of ceramic materials significantly improved their ability to mask underlying colors, highlighting the importance of material depth in achieving esthetic results. Furthermore, it was found that a thickness of at least 1 mm is required for zirconia ceramics (ZF) and 1.5 mm for VM ceramics to effectively mask an A5 background shade. In contrast, Celtra (CD) and Vita Suprinity (VS) ceramics did not demonstrate adequate color masking effectiveness at any of the tested thicknesses, indicating their drawbacks for clinical scenarios where optimal esthetic outcomes are essential.

## Author Contributions

**Seyede Mohaddeseh Hosseini Kalej:** investigation, project administration, visualization, writing – original draft preparation. **Behnaz Esmaeili:** data curation, supervision, writing – review and editing. **Ghazaleh Ahmadi Zenouz:** conceptualization, methodology, supervision, writing – review and editing. **Hemmat Gholinia:** formal analysis, methodology, writing – original draft preparation.

## Funding

The authors have nothing to report.

## Consent

The authors have nothing to report.

## Conflicts of Interest

The authors declare no conflicts of interest.

## Data Availability

The data that support the findings of this study are available from the corresponding author upon reasonable request.
